# Maternal Depressive Symptoms in the First Year after Childbirth Predict Long-Term Developmental Risks in Sons and Daughters

**DOI:** 10.3390/ijerph21030264

**Published:** 2024-02-24

**Authors:** Linda S. Pagani, Kianoush Harandian, Beatrice Necsa, Marie-Josée Harbec, George M. Tarabulsy

**Affiliations:** 1 School of Psycho-Education, University of Montreal, Montreal, QC H3C 3J7, Canada; kianoush.harandian@umontreal.ca (K.H.); beatrice.necsa@umontreal.ca (B.N.); 2 Sainte-Justine’s Pediatric Hospital Research Center, University of Montreal, Montreal, QC H3T 1C5, Canada; 3 School Environment Research Group, University of Montreal, Montreal, QC H3C 3J7, Canada; 4 Specialized Scientific Advisor, Institut National de Santé Publique du Québec, Montreal, QC H2P 1E2, Canada; 5 School of Psychology, Université Laval, Quebec, QC G1V 0A6, Canada

**Keywords:** maternal depressive symptoms, child development, psycho-social well-being, longitudinal analyses

## Abstract

Under-diagnosed and thus under-treated, maternal depression remains the most common complication of childbearing. Varying symptoms suggest persistence up to more than a decade following childbirth. This implies distinct vulnerabilities for the physical and emotional care of children. Using a prospective-longitudinal sex-stratified birth cohort of 2120 infants, we examined the relationship between early maternal depression symptoms and subsequent child psycho-social and relational characteristics. Mothers self-reported the severity and frequency of depressive symptoms 5 months after childbirth. Parents, teachers, and target participants reported on child mental health and relationships with adults, from kindergarten to tenth grade. A series of least-squares regressions were estimated, while controlling for pre-existing/concurrent child and family confounds. Both sons and daughters of mothers with more depressive symptoms were at risk of experiencing greater psycho-social impairment, classroom rule defiance, difficult relationships with teachers, less enjoyable mealtimes (age 6 years) and sleep, and coercive or inconsistent parenting practices in childhood and adolescence. For boys, these prospective associations were mostly consistent through ages 12 and 15 years. Girls also experienced more problematic interactions through to age 15 years. This study provides observations of distinct long-term vulnerabilities for sons and daughters in association with early maternal distress at important transitional periods of development in early, middle, and later childhood.

## 1. Introduction

Care of mothers begets care of children. Due to a confluence of biological and contextual factors, mental disorders remain the most common persistent complication of childbearing [1]. Affecting one in five women, maternal depression is both under-diagnosed and thus under-treated, especially in low- and middle-income countries [2]. The functional impairment associated with symptoms generates enduring social–economic costs to individuals, families, and communities [3,4].

The World Health Organization has underscored an urgent millennial goal for community-based knowledge in early identification and management of maternal mental health disorders and now offer clear care-giver guidelines [5,6]. A suggested worldwide healthcare standard is to regularly ask mothers about their experiences of coping with negative emotions (sadness, irritability, and anxiety) at each postnatal contact. They should be asked about daily tasks, social support, and remarkable family life events [7]. Family members should also be included by healthcare providers to assess any mental health changes that are considered atypical [1].

Screening begins with an assessment of the range of typical experiences with depressive symptoms during the first year after birth [8]. Depressed mood and loss of interest/motivation must be accompanied by several other symptoms over a two-week period, such as disturbances in sleep and/or appetite; feelings of worthlessness and/or guilt; diminished concentration; irritability; anxiety; and/or thoughts of suicide [5]. Functional impairment varies with symptomatology and phenotypically differs across women [9,10]. For some, even a narrow range of symptoms can handicap social and occupational functioning [5]. This is important for several reasons. First, there is reasonable concern for morbidity and mortality, especially when symptoms are endured past the first 5 months. Significant symptoms’ intensity should be prioritized for treatment because of the associated risks related to caregiving [11,12]. Second, varying levels of key depressive symptoms are likely to persist up to more than a decade after childbirth [13,14,15,16].

Indeed, signs of emotional distress in mothers forecast child outcomes [8]. Depressive symptoms generate disadvantage in neurodevelopmental milestones in the preschool years [17]. In a systematic review of 122 studies (out of 3712 candidate studies from 2005 to 2016), Slomian and her colleagues [18] synthetized bio-psycho-social development risks for child well-being from birth to age 3 years. Two other reviews of 29 studies documented similar negative associations with mother–child attachment and child cognitive and behavioral functioning [12,19]. A population-based Canadian birth cohort study of children born between 2005 and 2016 found that children exposed to maternal depressive symptoms in the first year of life had increased risk for difficulties in social competence, physical health and well-being, and emotional maturity by age 5 years [20]. Early childhood vulnerability potentially snowballs into adult risks [13].

There is a theoretical explanation to this nexus. Maternal depressive symptoms after birth have been viewed as the result of the activation of inflammatory pathways associated with perinatal demands [21]. This may impact child vulnerability to stress and subsequent risk of child psychopathology via hypothalamic–pituitary–adrenal axis functioning [22,23]. Classic reactivity models of “allostatic load” characterize less than optimal infant caregiving through the unbalanced interplay between maternal depressive symptoms, maternal cortisol secretion and child reactivity, and unmet emotional and physical needs [24]. Prolonged exposure to negative life experiences in infancy predicts less optimal glucocorticoid signaling and cortisol responses to daily stressors by adolescence [25]. Maternal and child reactivity are thus linked with negative reciprocal interactions, which involve bonding, breast-feeding, and warmly engaged and responsive care [18].

Studies vary in the techniques that measure symptoms versus a clinical diagnosis, as shown in the most stringent of literature reviews [12,18,19]. Compared to discrete diagnostic measurement, screening instruments offer more person-centered and continuous approaches [13,17]. Sensitivity by symptom intensity might better detect degrees of functional impairment in caregiving and parenting [1,22]. Varying the confound control across studies also reduces confidence in interpretations. Thus, pre-existing and concurrent factors control in a longitudinal design could maximize internal and external validity [26]. Finally, the majority of studies has relegated child sex as a nuisance variable that requires confound control. This disregards the influences of gender-based vulnerabilities due to biological and contextual influences in parent–child interactions [27,28]. Real life is not a gender-neutral experience. First, mothers of male infants have greater odds of postpartum depression likely attributable to inflammatory pathways during sex-specific gestation [21]. Second, sons of mothers experiencing distress during infancy and early childhood have been found to be particularly vulnerable, showing both long-term internalizing and externalizing behavior in childhood, when compared with daughters [28]. Because boys and girls have different biological and contextual influences and expectations, we argue that maternal depressive symptoms represent a distinct experience, thus requiring that we treat the two sexes as separate populations [29].

The purpose of this paper is to examine the association between maternal depressive symptoms in the first year after childbirth and subsequent psycho-social development from kindergarten entry to high school. Using a longitudinal population-based birth cohort design, we aim to examine whether maternal depressive symptoms 5 months after childbirth predict risks for key psychological and social behaviors from ages 6 to 15 years in typically developing boys and girls. Based on the concept of allostatic load and its recurrent associated chances of disorganized mutual reactivity in the dyadic mother–infant relationship, we expect that maternal depressive symptoms will predict enduring child risks. That is, we expect that more severe maternal depressive symptoms risk unmet physical and emotional infant needs, which generate enduring negative reactivity to perceived stress in the growing child and thus predict long-term problematic behaviors, negative interactions with peers and adults, and fewer opportunities to partake in developmentally appropriate activities.

## 2. Methods

### 2.1. Participants

The Quebec Longitudinal Study of Child Development (QLSCD), coordinated by the Institut de la Statistique du Québec, is made up of a birth cohort sample consisting of 2837 newborns born between 1997 and 1998. The participants were randomly selected through the Quebec Master Birth registry from remote and non-remote administrative regions of the health ministry. Participants residing in aboriginal territories or born before 24 weeks or after 42 weeks of gestation were excluded. The number of participants recruited from each sampling unit was determined by the number of births in the sampled regions during the previous year. From the original sample, 93 infants were deemed ineligible and 172 were untraceable. Of the 2572 eligible participants, 14 were unreachable and 438 refused to participate. In the early childhood phase, informed consent was obtained from the parents, after which informed consent was obtained from parents, children, and teachers in school-age years. Data from participants was collected through home interviews regarding parental (maternal and paternal), family, child, and environmental characteristics. The 5-month-old infants with complete maternal depressive symptoms data (*n* = 2120) were retained (50.7% boys). Outcomes were selected from data collection waves, which occurred at ages 6, 12, and 15 years.

### 2.2. Predictor Variable: Maternal Depressive Symptoms (Age 5 Months)

Depressive symptoms were measured with a valid and reliable abridged version of the Center for Epidemiologic Studies Depression Scale as reported by mothers themselves (13 items: my appetite was poor; I could not shake off the blues; I had trouble keeping my mind on what I was doing; I felt depressed; I felt that everything I did was an effort; I felt hopeful about the future (reverse-coded (RC)); my sleep was restless; I was happy (RC); I felt lonely; I enjoyed life (RC); I had crying spells; I felt that people disliked me; I have felt scared or panicky for no very good reason; 𝛂 = 0.81 for sons and 𝛂 = 0.78 for daughters) [30,31]. Mothers reported the frequency of each of these symptoms in the last week on a Likert scale with response options including 1 (rarely or never (less than 1 day)), 2 (some or a little of the time (1–2 days)), 3 (occasionally or a moderate amount of time (3–4 days)), and 4 (most or all the time (5–7 days)). Higher values indicate more severe maternal depression symptoms.

### 2.3. Outcome Variables: Psychological Outcomes (Ages 6, 12, and 15 Years)

Psycho-social adjustment. At ages 6 and 12 years, teachers reported child social adjustment in the past 6 months using three factors from the Social Behavior Questionnaire [32]: physical aggression (10 items: child got into fights; encouraged other children to pick on a particular child; reacted in an aggressive manner when teased; tried to dominate other children; reacted in an aggressive manner when contradicted; scared other children to get what she/he wanted; when someone accidentally bumped him/her, he/she started a fight; he/she reacted with anger and fighting; physically attacked people, hit, bit, or kicked other children; reacted in an aggressive way when something was taken away from he/her; 𝛂_6years_ = 0.92, 𝛂_12years_ = 0.90), oppositional behavior (4 items: was defiant or refused to comply with adult’s requests or rules; did not seem to feel guilty after misbehaving; punishment did not change his/her behavior; had temper tantrums or hot temper; 𝛂_6years_ = 0.81, 𝛂_12years_ = 0.83), and victimization (3 items: was made fun of by other children; was hit or pushed by other children; was called names by other children; 𝛂_6years_ = 0.74, 𝛂_12years_ = 0.73). At age 15 years, children self-reported on victimization (6 items: someone called me names, insulted me or said mean things to me; someone did not let me be part of his or her group when I wanted to; someone pushed, shoved, hit or kicked me; someone said bad things behind my back to other students; someone made fun of me, laughed at me; I was «taxed» by other students (someone made me pay them or give them something so they would leave me alone); 𝛂_15years_ = 0.82). Items were rated as 1 (never), 2 (rarely (once or twice)), 3 (often (about once a week on average)), and 4 (very often (more than once a week on average)) on a Likert scale. Parents and children reported on emotional distress at ages 6 and 12 years respectively (7 items: cried a lot; had trouble enjoying him/herself; was worried; was nervous, high-strung or tense; was too fearful or anxious; was not as happy as other children; seemed to be unhappy or sad; 𝛂_6years_ = 0.69, 𝛂_12years_ = 0.79). Response options for all items included 1 (never or not true), 2 (sometimes or somewhat true), and 3 (often or very true) on a Likert scale. Higher values indicate a higher intensity of emotional distress. With the exception of the emotional distress scale, all psychological adjustment scales were transformed on a scale from 0 to 10 using the mean score, where a higher score indicates a more problematic behavior.

Sleep length. The mother reported the amount of sleep (hours and minutes) the child has on an average night at ages 6, 12, and 15 years.

### 2.4. Outcome Variables: Social Outcomes (Ages 6, 12, and 15 Years)

Respect of rules. Teachers reported on the child’s respect of rules based on items from the Early Development Instrument^33^ at ages 6 and 12 years (8 items: follows rules; follows instructions; has respect for the property of others; has self-control; has respect for other adults; has respect for other youth; accepts responsibility for own actions; takes care of material; 𝛂_6years_ = 0.82, 𝛂_12years_ = 0.93). Responses to these items varied on a Likert scale of 1 (never), 2 (rarely), 3 (sometimes), 4 (often), and 5 (always). For each scale, the mean was calculated and converted to a scale ranging from 0 to 10.

Student–teacher relationships. At age 6 years, conflictual relationship with the child comprised 6 items (raised my voice, scold or yell at him/her; how often I have to discipline him/her repeatedly for the same thing; child tends to have more emotional and behavioral problems than other boys or girls his/her age; this child and I always seem to be struggling with each other; dealing with this child drains my energy; when this child is in a bad mood, I know we’re in for a long and difficult day; 𝛂_6years_ = 0.85) [33]. At age 12 years, sixth-grade teachers reported their relationship with the child (4 items: this child and I always seem to be struggling with each other; this child easily becomes angry with me; dealing with this child drains my energy; when this child is in a bad mood, I know we’re in for a long and difficult day; 𝛂_12years_ = 0.87) [34]. At age 15 years, youths self-reported on relationships with teachers (4 items: I get easily angry at teachers; it takes a lot of energy for a teacher to deal with me; I find it difficult to get along with teachers; I don’t feel respected by teachers; 𝛂_15years_ = 0.79) [34]. Items were rated on a Likert scale including 1 (not at all or does not apply), 2 (not really), 3 (neutral/not sure), 4 (applies somewhat), and 5 (a lot or definitely applies). The mean of the item scores was calculated and restructured on a scale from 0 to 10. Higher values indicate a more conflictual relationship.

Parental practices. (A) Consequential interactions with the child. At ages 6, 12, and 15 years, parents reported on how consistently the child is given consequences in the past 12 months (7 items: when you ask the child to do something, what portion of time do you check for compliance; if told child they would get punished if they did not stop doing something, and kept doing it, how often did you punish the child; how often did the child get away with things that you felt should have been punished; how often was the child able to get out of a punishment when you really had set his/her mind to it; when you had disciplined the child, how often did he/she ignore the punishment?; when child broke the rules or did things that they weren’t supposed to, how often did you ignore it; when child broke the rules or did things that they weren’t supposed to, how often did you take away privileges or sent them in their room; 𝛂_6years_ = 0.67, 𝛂_12years_ = 0.58, 𝛂_15years_ = 0.60) [35]. The Likert response range was 1 (never), 2 (rarely or less than half the time), 3 (sometimes or about half the time), 4 (often or more than half the time), and 5 (always or all the time). For all items, the Likert scale is inverted for a higher score to indicate a greater tendency to apply the same discipline rules for behaviors each time. (B) Coercive interactions with the child. At ages 6, 12, and 15 years, the parent reported on their negative interactions with the child in the past 12 months (7 items: you grab firmly or shake the child when they were being difficult; get angry with the child for saying or doing something they were not supposed to; hit the child when they were being difficult; get angry when you were punishing the child; have to discipline the child repeatedly for the same thing; raise your voice, scold or yell at the child when they broke the rules; use physical punishment toward the child when they broke the rules; 𝛂_6years_ = 0.74, 𝛂_12years_ = 0.68, 𝛂_15years_ = 0.59). The Likert scales response options were as follows: 1 (never), 2 (about once a month or less), 3 (about once per 2 weeks), 4 (about once a week), 5 (a few times a week), 6 (one or two times a day), and 7 (many times each day).

Family meal environment quality. Parent-reported family meal environment quality scale used by Pagani et al. [36] measured mealtime enjoyment and communication at age 6 years.

Bedroom screen. The child reported at age 12 years if there was the presence of a television in the bedroom. Response options were 0 (no) or 1 (yes).

### 2.5. Confound Control Variables (Ages 5 Months to 15 Years)

To rule out pre-existing confounding variables between maternal depressive symptoms and later psycho-social outcomes, child-specific and family characteristics were identified based on previous literature and statistical analyses. From the National Institute of Mental Health-Diagnostic Interview Schedule [37], we obtained parent-reported mother antisocial antecedents (9 items: 0 = below the median, 1 = above the median; 𝛂 = 0.51) and father antisocial antecedents at 5 months (8 items: 0 = below the median, 1 = above the median; 𝛂 = 0.62); family configuration at 5 months (0 = intact, 1 = non-intact); family income at 5 months (0 = sufficient income, 1 = insufficient income); maternal education at 5 months (0 = finished high school, 1 = did not finish high school); paternal education at 5 months (0 = finished high school, 1 = did not finish high school); gestational smoking or substance use at 5 months (0 = no substance use, 1 = any substance use); neurocognitive skills assessed through the “1, 2, 3 Hands Game”, an adapted version of the Imitation Sorting Task, administered by research assistants at 5 months [38]; and child temperament problems using the Infant Characteristics Questionnaire (ICQ) at age 1.5 years as reported by the father (10 items: 0 = below the median, 1 = above the median; 𝛂 = 0.76) [39]. Parent-reported child screen time (0 = below 2 h/day, 1 = above 2 h/day) and family dysfunction were measured using items from the McMaster Family Assessment Device [40] (7 to 9 items: 0 = below the median, 1 = above the median; 𝛂_6years_ = 0.82, 𝛂_12years_ = 0.85, 𝛂_15years =_ 0.83), and both were controlled for, concurrently, at ages 6, 12, and 15 years.

### 2.6. Data Analytic Procedures

This study required data from several sources and waves for predictors, outcomes, and potential confounders from ages ranging from 5 months to 15 years. Incomplete data were expected, as with any longitudinal study. An attrition analysis compared retained participants with complete and incomplete data on control variables. Using chi-squared tests, significant differences were found. Compared to girls with complete data, girls with incomplete data belonged more often to non-intact families (X^2^ (1, *n* = 1040) = 4.569, *p* < 0.05) and had mothers and fathers with lower education (X^2^ (1, *n* = 1045) = 5.756, *p* < 0.05, and X^2^ (1, *n* = 966) = 6.365, *p* < 0.01, respectively). Boys with incomplete data had mothers with a greater antisocial behavior history (X^2^ (1, *n* = 1043) = 4.674, *p* < 0.05), were more subjected to gestational smoking or substance use (X^2^ (1, *n* = 1079) = 8.478, *p* < 0.01), were more from families with insufficient income (X^2^ (1, *n* = 1068) = 6.024, *p* < 0.01), and had mothers and fathers who were less likely to have finished high school (X^2^ (1, *n* = 1081) = 3.832, *p* < 0.05, and X^2^ (1, *n* = 990) = 8.123, *p* < 0.01, respectively). Moreover, these boys belonged to families with greater dysfunction at 6 years (X^2^ (1, *n* = 731) = 7.340, *p* < 0.01). Multiple imputation was used to correct for response and attrition bias [41].

Our analyses employed a sex-stratified approach, thus treating boys and girls as separate populations as they experience risk and protective factors differently due to distinct biological and contextual influences [42]. We first estimated a series of least-squares regressions in which maternal depressive symptoms at age 5 months were linearly regressed on substantively or theoretically related child and family characteristics from ages 5 months to 15 years. We then estimated a series of least-squares regressions in which several indicators of psycho-social well-being at ages 6, 12, and 15 years were predicted by maternal depressive symptoms at age 5 months (SPSS v.26, IBM, Armonk, NY, USA). To best ensure an unbiased estimation of these presumed associations, we adjusted for pre-existing and concurrent child and family characteristics. That is, for each outcome, the predictor and confound controls were entered simultaneously for a fully controlled model, where DR_iages6–15years_ represents child well-being risks; MDS_iage5months_ represents exposure to maternal depressive symptoms at age 5 months; and FAM_i_ and CHILD_i_ represent, respectively, the family and child control variables that are pre-existing to the MDS predictor variable or concurrent with the DR outcome variable for each individual child_i_. Finally, a_1_ and e_i_ represent the constant and the stochastic error terms, respectively.

DR_iages6–15years_ = a1 + ß1 MDS_iage5months_ + γ1 CHILD_i_ + γ2 FAM_i_ + e_i_

## 3. Results

Table 1 provides descriptive statistics stratified by sex for the predictor, outcome, and control variables. At age 5 months, mothers of sons had a mean score of 1.42 (on 10) on the depressive symptoms scale. As for daughters, mothers had a mean score of 1.36 on the depressive symptoms scale.

Table 2 documents the relationship between the baseline and concurrent control variables from ages 5 months to age 15 years and maternal depressive symptoms at age 5 months. For boys and girls, maternal depressive symptoms was associated with higher mother antisocial behavior (β_boys_ = 0.095, *p* ≤ 0.01, 95% confidence interval (CI) from 0.11 to 0.49; β_girls_ = 0.144, *p* ≤ 0.001, 95% CI from 0.24 to 0.60) and insufficient family income (β_boys_ = 0.129, *p* ≤ 0.001, 95% CI from 0.21 to 0.62; β_girls_ = 0.145, *p* ≤ 0.001, 95% CI from 0.24 to 0.65) at age 5 months, and with higher family dysfunction at age 6 years for boys (β = 0.099, *p* ≤ 0.01, 95% CI from 0.10 to 0.45) and at age 15 years for girls (β = 0.081, *p* ≤ 0.05, 95% CI from 0.04 to 0.38). Moreover, for boys, living in a non-intact family at age 5 months was positively associated with maternal depressive symptoms (β = 0.061, *p* ≤ 0.05, 95% CI from 0.00 to 0.44).

Table 3 reports the adjusted relationship between maternal depressive symptoms at age 5 months and teacher- and parent-reported psycho-social outcomes at age 6 years, stratified by sex. For both boys and girls, higher maternal depressive symptoms predicted higher levels of physical aggression (β_boys_ = 0.118, *p* ≤ 0.001, 95% CI from 0.06 to 0.20; β_girls_ = 0.142, *p* ≤ 0.001, 95% CI from 0.08 to 0.20), oppositional behavior (β_boys_ = 0.167, *p* ≤ 0.001, 95% CI from 0.13 to 0.29; β_girls_ = 0.140, *p* ≤ 0.001, 95% CI from 0.09 to 0.24), victimization (β_boys_ = 0.099, *p* ≤ 0.001, 95% CI from 0.03 to 0.12; β_girls_ = 0.107, *p* ≤ 0.001, 95% CI from 0.04 to 0.14), and emotional distress (β_boys_ = 0.125, *p* ≤ 0.001, 95% CI from 0.09 to 0.27; β_girls_ = 0.181, *p* ≤ 0.001, 95% CI from 0.18 to 0.36). For both boys and girls, a higher maternal depressive symptoms predicted lower levels of the child’s respect of rules (β_boys_ = −0.163, *p* ≤ 0.001, 95% CI from −0.23 to −0.10; β_girls_ = −0.134, *p* ≤ 0.001, 95% CI from −0.20 to −0.07) and family meal environment quality (β_boys_ = −0.099, *p* ≤ 0.001, 95% CI from −0.03 to −0.01; β_girls_ = −0.068, *p* ≤ 0.01, 95% CI from −0.03 to 0.00). Furthermore, for both boys and girls, higher maternal depressive symptoms predicted higher levels of conflictual student–teacher relationships (β_boys_ = 0.141, *p* ≤ 0.001, 95% CI from 0.09 to 0.23; β_girls_ = 0.095, *p* ≤ 0.001, 95% CI from 0.04 to 0.17) and parental coercive interactions with the child (β_boys_ = 0.145, *p* ≤ 0.001, 95% CI from 0.06 to 0.14; β_girls_ = 0.141, *p* ≤ 0.001, 95% CI from 0.06 to 0.15). For girls only, higher maternal depressive symptoms predicted lower levels of parental consequential interactions with the child (β = −0.074, *p* ≤ 0.05, 95% CI from −0.13 to −0.01). 

Table 4 reports the adjusted relationship between maternal depressive symptoms at age 5 months and psycho-social outcomes at age 12 years, stratified by sex, as reported by teachers, parents, and children. For both boys and girls, higher maternal depressive symptoms predicted lower levels of parental consequential interactions with the child (β_boys_ = −0.062, *p* ≤ 0.05, 95% CI from −0.13 to 0.00; β_girls_ = −0.079, *p* ≤ 0.01, 95% CI from −0.17 to −0.02). For boys, higher maternal depressive symptoms predicted higher levels of physical aggression (β = 0.068, *p* ≤ 0.05, 95% CI from 0.01 to 0.12), oppositional behavior (β = 0.059, *p* ≤ 0.05, 95% CI from 0.00 to 0.15), victimization (β = 0.062, *p* ≤ 0.05, 95% CI from 0.00 to 0.14), emotional distress (β = 0.074, *p* ≤ 0.05, 95% CI from 0.02 to 0.21), conflictual student–teacher relationships (β = 0.065, *p* ≤ 0.05, 95% CI from 0.01 to 0.14), and parental coercive interactions with the child (β = 0.081, *p* ≤ 0.01, 95% CI from 0.01 to 0.07). Furthermore, higher levels of maternal depressive symptoms predicted lower levels of child’s respect of rules (β = −0.077, *p* ≤ 0.01, 95% CI from −0.12 to −0.02). For girls, higher maternal depressive symptoms was positively associated with having a television in the bedroom (β = 0.098, *p* ≤ 0.001, 95% CI from 0.01 to 0.06).

Table 5 reports the adjusted relationship between maternal depressive symptoms at age 5 months and bio-psycho-social outcomes at age 15 years, stratified by sex, as reported by arents and children. For both boys and girls, higher maternal depressive symptoms predicted shorter sleep length (β_boys_ = −0.161, *p* ≤ 0.001, 95% CI from −0.11 to −0.05; β_girls_ = −0.105, *p* ≤ 0.001, 95% CI from −0.10 to −0.02) and lower levels of parental consequential interactions with the child (β_boys_ = −0.061, *p* ≤ 0.05, 95% CI from −0.13 to 0.00; β_girls_ = −0.087, *p* ≤ 0.01, 95% CI from −0.18 to −0.03). For boys, higher maternal depressive symptoms predicted higher levels of victimization (β = 0.117, *p* ≤ 0.001, 95% CI from 0.05 to 0.17) and parental coercive interactions with the child (β = 0.081, *p* ≤ 0.01, 95% CI from 0.10 to 0.07). Interestingly, for girls, higher maternal depressive symptoms predicted a less conflictual student–teacher relationship (β = −0.085, *p* ≤ 0.01, 95% CI from −0.22 to −0.03).

## 4. Discussion

As the twig is bent, the tree shall grow. Past research suggests that maternal psychopathology influences the quality of mother–child and caregiving interactions, and, in turn, forecasts child vulnerability [1,2,7]. Our findings provide observations of distinct vulnerabilities for sons and daughters at important transitional periods of development at kindergarten, middle school, and mid-point of high school.

Kindergarten represents the beginning of formal schooling [43]. Both cognitive and non-cognitive school readiness skills ultimately influence long-term achievement, lifestyle, and ultimately human capital [44]. According to teachers, children of mothers reporting sadness, irritability, and lack of interest, pleasure, and energy 5 months after childbirth experienced more subsequent vulnerability at kindergarten, compared to their same-sex counterparts. More specifically, they had a greater risk of experiencing fear, sadness, and being physically aggressive and oppositional in the kindergarten setting. Such children were less likely to consider and respect others and their property, according to kindergarten teachers. Teachers also noted greater chances of being harassed, excluded, or insulted by classmates. It is not surprising that maternal depressive symptoms forecasted a conflictual relationship with kindergarten teachers. Teachers also reported greater risks of scolding, disciplining, or yelling at children of such mothers for repetitive problematic behaviors. The mothers themselves reported less optimal social interactions with their sons and daughters. For example, they were less likely to enjoy family meals as a time to communicate positively and were more likely to engage in coercive and negative interactions with their children.

Mothers who reported depressive symptoms at 5 months were more likely to use consequential parenting with girls at age 6 years. As such, they used more compliance monitoring, discipline, and contingent punishment with daughters. Taken together, this parenting approach by age 12 years may have offset some long-term problem behavior risks. Maternal depressive symptoms predicted having a child bedroom screen as well, which allows for private internet access and unregulated use and content. Bedroom screens at this age enhance temptation to spend more isolated discretionary time on entertainment rather than schoolwork [45]. Children with bedroom screens also sleep less. By age 15 years, daughters were more likely to report compromised sleep length and conflictual student–teacher relationships. Mothers continued to report consequential discipline and compliance checks with daughters in comparison to their same-sex counterparts with no history of maternal depressive symptoms.

For boys, the associations between maternal depressive symptoms and physical aggression, oppositional behavior, victimization by classmates, and emotional distress risks endured to the end of sixth grade, according to teachers. In this context, associations between maternal depressive symptoms and lack of respect for the rules and conflictual teacher–student relationships persisted as well. Sixth-grade teachers were more likely to report negative interactions with boys for their chronic problematic behaviors. It is not surprising that parents of such boys expressed having a harder time. Indeed, although they reported compliance checks and discipline contingencies, maternal depressive symptoms predicted more negative, harsh interactions with sons. By the mid-point of high school, sons of mothers with more depressed symptoms self-reported sleeping less and an increased risk of being victimized by schoolmates. This corroborates previous victimization reports by kindergarten and sixth-grade teachers and is not surprising, given their developmental risk trajectories of turbulent behavior since kindergarten. Although they reported increases in consequential discipline, parents were more likely to resort to more authoritarian and harsh interactions with adolescent sons by age 15 years.

This study is not without limitations. First, although correlation does not imply causation, maternal depressive symptoms might have predicted habitual negative interactions that influence a family climate of chronic adversity that further reinforce negative dyadic relations. Second, although the findings are above and beyond the influence of nuisance variables and omitted variable bias, we only tested the predictive value of depressive symptoms 5 months after childbirth. This study highlights the chronic power of early maternal symptoms. It has several strengths, especially the large sample, the abundant confound control variables, and the focus on critical developmental periods for outcomes of sons and daughters.

## 5. Conclusions

Maternal depression remains under-diagnosed and under-treated and is often persistent, thus requiring active clinical assessment and intervention to diminish populational risk [10,22]. It is more common in contexts where physical and emotional support for mothers is limited [5,6]. This likely affects cross-cultural and situational generalizability. Our findings, generated based on maternal states at one key point in early infancy, clearly speak to the extant literature. Moreover, they add a tale of developmental continuity using exhaustive control of confounds and competing pre-existing and concurrent explanations. Affecting stress and immune biomarkers, depressive symptoms in infancy create a unique environment that is not conducive to optimal development of both mothers and children [7,15]. The associated general irritable mood in such mothers likely generates a needs mismatch between parent and offspring, which, in turn, fosters habitual emotional dysregulation and chronic perceived stress response routines in the dyad from early childhood onward. Once coercive and counter-coercive transactions become habitual between parent and child, there are increased chances of diffusion effects of family adversity to other contexts, such as the classroom and school environment. Functional impairment in children is often expressed with negative behavior, such as irritability and underachievement [7]. The treatment of psycho-social difficulties in childhood and adolescence must consider parental mental health history and its long-term influence on the parent and child. Indeed, considering a life history approach to relationships may impact the potential effectiveness of potential interventions implemented during childhood and adolescence [27].

## Figures and Tables

**Table 1:** Descriptive statistics for study variables.

		Boys			Girls	
Variables	M (SD)	Categorical Variables (%)	Range	M (SD)	Categorical Variables (%)	Range
Predictor (5 mo)						
Maternal depressive symptoms ^2^	1.42 (1.39)		0.00–10.00	1.36 (1.27)		0.00–10.00
Outcomes (6 years)						
Sleep length ^2^	10.32 (0.71)		6.00–13.00	10.38 (0.72)		7.00–13.00
Physical aggression ^1^	1.85 (1.52)		0.00–10.00	1.40 (1.24)		0.00–10.00
Oppositional behavior ^1^	2.27 (1.74)		0.00–10.00	1.68 (1.52)		0.00–10.00
Victimization ^1^	1.33 (1.03)		0.00–10.00	1.12 (1.08)		0.00–10.00
Emotional distress ^2^	10.48 (2.03)		7.00–21.00	10.46 (1.88)		7.00–21.00
Respect of rules ^1^	8.09 (1.41)		0.00–10.00	8.57 (1.26)		0.00–10.00
Student–teacher relationship ^1^	2.24 (1.55)		0.00–10.00	1.72 (1.37)		0.00–10.00
Consequential interactions with the child ^2^	7.21 (1.15)		0.00–10.00	7.12 (1.18)		0.00–10.00
Coercive interactions with the child ^2^	2.65 (0.97)		0.00–10.00	2.54 (0.96)		0.00–10.00
Family meal environment quality ^2^	3.42 (0.29)		1.00–4.00	3.42 (0.28)		1.00–4.00
Outcomes (12 years)						
Sleep length ^2^	9.19 (0.67)		5.00–13.00	9.17 (0.65)		6.00–12.00
Physical aggression ^1^	1.42 (1.25)		0.00–10.00	1.01 (0.94)		0.00–10.00
Oppositional behavior ^1^	2.27 (1.80)		0.00–10.00	1.66 (1.55)		0.00–10.00
Victimization ^1^	1.82 (1.54)		0.00–10.00	1.44 (1.36)		0.00–10.00
Emotional distress ^3^	10.29 (2.14)		7.00–21.00	10.76 (2.32)		7.00–21.00
Respect of rules ^1^	7.87 (1.19)		0.00–10.00	8.46 (1.10)		0.00–10.00
Student–teacher relationship ^1^	1.80 (1.56)		0.00–10.00	1.34 (1.33)		0.00–10.00
Consequential interactions with the child ^2^	6.93 (1.44)		0.00–10.00	6.78 (1.54)		0.00–10.00
Coercive interactions with child ^2^	1.50 (0.70)		0.00–10.00	1.37 (0.72)		0.00–10.00
Bedroom screen ^3^ 1 = Has a TV in the bedroom		37.6			31.7	
Outcomes (15 years)						
Sleep length ^2^	8.42 (0.67)		6.00–12.00	8.36 (0.74)		4.00–12.00
Victimization ^3^	1.45 (1.28)		0.00–10.00	1.36 (1.24)		0.00–10.00
Student–teacher relationship ^3^	2.67 (1.86)		0.00–10.00	2.27 (1.86)		0.00–10.00
Consequential interactions with the child ^2^	6.97 (1.54)		0.00–10.00	7.05 (1.68)		0.00–10.00
Coercive interactions with the child ^2^	1.92 (0.93)		0.00–10.00	1.77 (0.98)		0.00–10.00
Control variables						
Mother antisocial behavior (5 mo) ^2^ 1 = Above the median		26.2			24.8	
Father antisocial behavior (5 mo) ^2^ 1 = Above the median		47.1			44.2	
Family configuration (5 mo) ^2^ 1 = Non-intact		17.9			19.1	
Family income (5 mo) ^2^ 1 = Insufficient		24.8			21.6	
Maternal education (5 mo) ^2^ 1 = Did not finish high school		15.4			15.6	
Paternal education (5 mo) ^2^ 1 = Did not finish high school		24.2			25.1	
Gestational smoking or substance use (5 mo) ^2^ 1 = Any smoking or substance use		31.8			31.9	
Child neurocognitive skills (5 mo) ^4^ 1 = Below the median		48.1			48.4	
Child temperament problems (1.5 year) ^2^ 1 = Above the median		48.8			45.7	
Child screen time (6 years) ^2^ 1 = Above 2 h a day		64.4			60.8	
Child screen time (12 years) ^2^ 1 = Above 2 h a day		56.9			50.6	
Child screen time (15 years) ^2^ 1 = Above 2 h a day		87.8			80.3	
Family dysfunction (6 years) ^2^ 1 = Above the median		43.5			43.0	
Family dysfunction (12 years) ^2^ 1 = Above the median		42.7			38.8	
Family dysfunction (15 years) ^2^ 1 = Above the median		40.6			38.7	

Notes. ^1^ Teacher-reported. ^2^ Parent-reported. ^3^ Child self-reported. ^4^ Trained examiner. M = mean; SD = standard deviation; TV = television. Analyses corrected for attrition bias. Data were compiled from the final master file of the Quebec Longitudinal Study of Child Development (1998–2013), © Gouvernement du Québec, Institut de la statistique du Québec.

**Table 2:** Unstandardized regression coefficients (standard error) reflecting the adjusted relationship between baseline and concurrent child and family characteristics between ages 5 months and 15 years and maternal depressive symptoms at age 5 months.

Variables	*b* (SE)	
	Maternal Depressive Symptoms (5 mo) ^2^	
Sex	**0.07 (0.06)**	
	**Boys**	**Girls**
Mother antisocial behavior (5 mo) ^2^	0.30 (0.10) **	0.42 (0.09) ***
Father antisocial behavior (5 mo) ^2^	0.13 (0.09)	0.13 (0.08)
Family configuration (5 mo) ^2^	0.22 (0.11) *	−0.01 (0.10)
Family income (5 mo) ^2^	0.42 (0.11) ***	0.45 (0.11) ***
Maternal education (5 mo) ^2^	0.13 (0.13)	0.04 (0.12)
Paternal education (5 mo) ^2^	0.13 (0.11)	−0.02 (0.10)
Gestational smoking or substance use (5 mo) ^2^	−0.09 (0.09)	0.14 (0.09)
Child neurocognitive skills (5 mo) ^4^	−0.08 (0.08)	−0.01 (0.08)
Child temperament problems (1.5 years) ^2^	0.06 (0.08)	0.00 (0.08)
Child screen time (6 years) ^2^	0.09 (0.09)	0.04 (0.08)
Child screen time (12 years) ^2^	0.01 (0.09)	0.06 (0.08)
Child screen time (15 years) ^2^	−0.21 (0.13)	−0.01 (0.10)
Family dysfunction (6 years) ^2^	0.28 (0.09) **	0.11 (0.08)
Family dysfunction (12 years) ^2^	0.15 (0.10)	0.12 (0.09)
Family dysfunction (15 years) ^2^	0.08 (0.09)	0.21 (0.09) *

Notes. * *p* ≤ 0.05, ** *p* ≤ 0.01, and *** *p* ≤ 0.001. ^1^ Teacher-reported. ^2^ Parent-reported. ^3^ Child self-reported. ^4^ Trained examiner. Analyses corrected for attrition bias. Data were compiled from the final master file of the Quebec Longitudinal Study of Child Development (1998–2013), *©* Gouvernement du Québec, Institut de la statistique du Quebec.

**Table 3:** Unstandardized regression coefficients (standard error) reflecting the adjusted relationship between maternal depressive symptoms at age 5 months and psycho-social outcomes at age 6 years.

		Age 6 Years								
	Variables	Physical Aggression ^1^	Oppositional Behavior ^1^	Victimization ^1^	Emotional Distress ^2^	Respects Rules ^1^	Student–Teacher Relationship ^1^	Consequential Interactions with the Child ^2^	Coercive Interactions with the Child ^2^	Family Meal Environment Quality ^2^
**Boys**	Maternal depressive symptoms (5 mo) ^2^	0.13 (0.03) ***	0.21 (0.04) ***	0.07 (0.02) ***	0.18 (0.05) ***	−0.17 (0.03) ***	0.16 (0.04) ***	−0.04 (0.03)	0.10 (0.02) ***	−0.02 (0.01) ***
	Mother antisocial behavior (5 mo) ^2^	0.20 (0.11)	0.31 (0.12) **	0.22 (0.07) **	0.05 (0.15)	−0.22 (0.10) *	0.17 (0.11)	−0.08 (0.08)	0.14 (0.07) *	−0.04 (0.02) *
	Father antisocial behavior (5 mo) ^2^	0.19 (0.10) *	0.07 (0.11)	0.04 (0.06)	0.01 (0.13)	−0.11 (0.09)	0.13 (0.10)	−0.01 (0.07)	−0.04 (0.06)	0.00 (0.02)
	Family configuration (5 mo) ^2^	0.10 (0.13)	0.19 (0.15)	0.21 (0.09) *	0.08 (0.17)	−0.06 (0.12)	0.11 (0.13)	−0.07 (0.09)	−0.09 (0.03)	0.03 (0.02)
	Family income (5 mo) ^2^	−0.02 (0.12)	−0.11 (0.14)	0.08 (0.08)	0.17 (0.16)	0.18 (0.11)	−0.10 (0.12)	−0.09 (0.09)	−0.03 (0.08)	−0.02 (0.02)
	Maternal education (5 mo) ^2^	0.38 (0.14) **	0.54 (0.16) ***	0.28 (0.10) **	−0.34 (0.19)	−0.38 (0.13) **	0.39 (0.15) **	−0.26 (0.10) **	−0.14 (0.09)	0.06 (0.02) **
	Paternal education (5 mo) ^2^	0.09 (0.12)	0.07 (0.13)	−0.07 (0.08)	0.33 (0.16) *	−0.06 (0.11)	0.07 (0.12)	−0.35 (0.09) ***	0.03 (0.07)	−0.01 (0.02)
	Gestational smoking or substance use (5 mo) ^2^	0.10 (0.10)	−0.02 (0.12)	−0.14 (0.07) *	0.14 (0.14)	−0.17 (0.09)	−0.11 (0.10)	0.09 (0.08)	−0.04 (0.06)	0.02 (0.02)
	Child neurocognitive skills (5 mo) ^4^	0.10 (0.09)	0.05 (0.11)	0.01 (0.06)	0.07 (0.12)	0.08 (0.08)	0.04 (0.09)	0.14 (0.07) *	0.06 (0.06)	−0.01 (0.01)
	Child temperament problems (1.5 years) ^2^	0.04 (0.09)	−0.04 (0.11)	−0.01 (0.06)	0.31 (0.12) **	0.05 (0.08)	−0.04 (0.09)	0.04 (0.07)	0.10 (0.06)	−0.01 (0.01)
	Child screen time (6 years) ^2^	0.04 (0.10)	−0.12 (0.11)	0.13 (0.07) *	−0.12 (0.13)	−0.06 (0.09)	0.00 (0.10)	−0.21 (0.07) **	0.13 (0.06) *	−0.03 (0.02) *
	Family dysfunction (6 years)^2^	0.17 (0.09)	0.20 (0.11)	0.14 (0.06) *	0.48 (0.13) ***	−0.22 (0.09) **	0.09 (0.10)	−0.29 (0.07) ***	0.33 (0.06) ***	−0.35 (0.01) ***
	Adjusted R^2^	0.048 ***	0.061 ***	0.061 ***	0.045 ***	0.067 ***	0.035 ***	0.084 ***	0.063 ***	0.400 ***
**Girls**	Maternal depressive symptoms (5 mo) ^2^	0.14 (0.03) ***	0.17 (0.04) ***	0.09 (0.03) ***	0.27 (0.05) ***	−0.13 (0.03) ***	0.10 (0.03) **	−0.07 (0.03) *	0.11 (0.02) ***	−0.02 (0.01) **
	Mother antisocial behavior (5 mo) ^2^	0.29 (0.09) **	0.22 (0.11) *	0.07 (0.08)	0.17 (0.14)	−0.16 (0.09)	0.14 (0.10)	0.01 (0.09)	0.14 (0.07) *	−0.07 (0.02) ***
	Father antisocial behavior (5 mo) ^2^	0.13 (0.08)	0.14 (0.10)	0.06 (0.07)	0.03 (0.12)	−0.15 (0.08)	0.21 (0.09) *	−0.04 (0.08)	0.07 (0.06)	0.00 (0.02)
	Family configuration (5 mo) ^2^	0.08 (0.10)	0.08 (0.13)	0.06 (0.09)	−0.10 (0.16)	−0.02 (0.11)	0.06 (0.12)	−0.16 (0.10)	−0.10 (0.8)	−0.03 (0.02)
	Family income (5 mo) ^2^	−0.04 (0.11)	−0.06 (0.13)	0.18 (0.09) *	0.15 (0.16)	0.15 (0.11)	0.04 (0.12)	−0.10 (0.10)	0.03 (0.08)	−0.01 (0.02)
	Maternal education (5 mo) ^2^	0.28 (0.12) *	0.44 (0.15) **	0.33 (0.10) ***	−0.06 (0.18)	−0.20 (0.12)	0.32 (0.13) **	−0.22 (0.11) *	−0.09 (0.09)	0.06 (0.02) **
	Paternal education (5 mo) ^2^	−0.16 (0.10)	−0.12 (0.12)	−0.15 (0.09)	−0.17 (0.15)	−0.03 (0.10)	−0.19 (0.11)	−0.13 (0.09)	0.00 (0.08)	0.01 (0.02)
	Gestational smoking or substance use (5 mo) ^2^	0.11 (0.09)	0.05 (0.10)	−0.04 (0.07)	−0.03 (0.13)	−0.09 (0.09)	0.06 (0.09)	0.01 (0.08)	0.09 (0.07)	0.01 (0.02)
	Child neurocognitive skills (5 mo) ^4^	0.00 (0.08)	0.03 (0.09)	0.03 (0.07)	−0.01 (0.11)	0.04 (0.08)	0.10 (0.08)	0.06 (0.07)	0.04 (0.06)	0.01 (0.01)
	Child temperament problems (1.5 years) ^2^	0.17 (0.08) *	0.05 (0.09)	0.08 (0.07)	0.05 (0.11)	−0.12 (0.08)	0.14 (0.08)	−0.02 (0.07)	0.07 (0.06)	−0.01 (0.01)
	Child screen time (6 years) ^2^	0.00 (0.08)	−0.05 (0.10)	0.01 (0.07)	0.26 (0.12) *	0.08 (0.08)	0.12 (0.09)	−0.31 (0.07) ***	0.16 (0.06) **	−0.02 (0.01)
	Family dysfunction (6 years)^2^	−0.06 (0.08)	0.17 (0.04)	0.04 (0.07)	0.58 (0.12) ***	−0.06 (0.08)	−0.11 (0.09)	−0.37 (0.07) ***	0.26 (0.06) ***	−0.35 (0.01) ***
	Adjusted R^2^	0.050 ***	0.035 ***	0.034 ***	0.067 ***	0.031 ***	0.029 ***	0.083 ***	0.059 ***	0.409 ***

Notes. * *p* ≤ 0.05, ** *p* ≤ 0.01, and *** *p* ≤ 0.001. ^1^ Teacher-reported. ^2^ Parent-reported. ^3^ Child self-reported. ^4^ Trained examiner. Analyses corrected for attrition bias. Data were compiled from the final master file of the Quebec Longitudinal Study of Child Development (1998–2013), © Gouvernement du Québec, Institut de la statistique du Québec.

**Table 4:** Unstandardized regression coefficients (standard error) reflecting the adjusted relationship between maternal depressive symptoms at age 5 months and psycho-social outcomes at age 12 years.

		Age 12 Years								
	Variables	Physical Aggression ^1^	Oppositional Behavior ^1^	Victimization ^1^	Emotional Distress ^3^	Respect of Rules ^1^	Student–Teacher Relationship ^1^	Consequential Interactions with the Child ^2^	Coercive Interactions with the Child ^2^	Bedroom Screen ^3^
**Boys**	Maternal depressive symptoms (5 mo) ^2^	0.06 (0.03) *	0.08 (0.04) *	0.07 (0.04) *	0.12 (0.05) *	−0.07 (0.03) **	0.07 (0.04) *	−0.07 (0.03) *	0.04 (0.02) **	0.01 (0.01)
	Mother antisocial behavior (5 mo) ^2^	0.17 (0.09)	0.52 (0.13) ***	0.23 (0.011) *	0.06 (0.15)	−0.47 (0.08) ***	0.37 (0.11) ***	−0.27 (0.10) **	0.04 (0.05)	0.07 (0.03) *
	Father antisocial behavior (5 mo) ^2^	−0.08 (0.08)	0.14 (0.11)	0.06 (0.10)	0.10 (0.14)	−0.14 (0.07)	0.18 (0.10)	0.03 (0.09)	0.04 (0.05)	0.00 (0.03)
	Family configuration (5 mo) ^2^	0.11 (0.11) **	0.52 (0.15) ***	0.09 (0.13)	0.23 (0.18)	−0.23 (0.10) *	0.26 (0.13) *	−0.16 (0.12)	−0.04 (0.06)	0.04 (0.04)
	Family income (5 mo) ^2^	0.27 (0.10)	0.13 (0.14)	0.15 (0.12)	0.37 (0.17) *	0.03 (0.09)	0.18 (0.12)	−0.03 (0.11)	0.06 (0.06)	0.20 (0.04) ***
	Maternal education (5 mo) ^2^	0.06 (0.12)	−0.02 (0.16)	0.09 (0.15)	0.18 (0.20)	−0.25 (0.11) *	−0.19 (0.14)	−0.07 (0.13)	0.07 (0.07)	0.09 (0.04) *
	Paternal education (5 mo) ^2^	0.07 (0.10)	0.03 (0.14)	−0.20 (0.12)	0.12 (0.17)	0.16 (0.09)	0.04 (0.12)	−0.22 (0.11) *	−0.03 (0.06)	0.15 (0.04) ***
	Gestational smoking or substance use (5 mo) ^2^	0.16 (0.08)	0.27 (0.12) *	0.02 (0.10)	0.27 (0.14)	−0.22 (0.08) **	0.16 (0.10)	−0.44 (0.10) ***	0.10 (0.05) *	0.11 (0.03) ***
	Child neurocognitive skills (5 mo) ^4^	−0.03 (0.08)	−0.05 (0.11)	−0.15 (0.09)	−0.16 (0.13)	0.10 (0.07)	−0.21 (0.09) *	0.11 (0.09)	0.02 (0.04)	−0.02 (0.03)
	Child temperament problems (1.5 years) ^2^	−0.11 (0.08)	−0.39 (0.11) ***	−0.14 (0.10)	−0.16 (0.13)	0.25 (0.07) ***	−0.34 (0.09) ***	−0.11 (0.09)	0.00 (0.04)	0.00 (0.03)
	Child screen time (12 years) ^2^	0.00 (0.08)	0.18 (0.11)	−0.02 (0.10)	0.26 (0.13) *	−0.03 (0.07)	0.13 (0.10)	−0.22 (0.09) **	0.00 (0.04)	−0.02 (0.03)
	Family dysfunction (12 years) ^2^	0.06 (0.08)	0.17 (0.11)	−0.07 (0.10)	0.29 (0.13) *	−0.16 (0.07) *	0.16 (0.10)	−0.02 (0.09)	0.16 (0.04) ***	0.01 (0.03)
	Adjusted R^2^	0.031 ***	0.072 ***	0.012 *	0.039 ***	0.091 ***	0.057 ***	0.058 ***	0.026 ***	0.128 ***
**Girls**	Maternal depressive symptoms (5 mo) ^2^	0.01 (0.02)	0.00 (0.04)	0.01 (0.03)	0.04 (0.06)	−0.03 (0.03)	0.00 (0.03)	−0.10 (0.04) **	0.01 (0.02)	0.04 (0.01) ***
	Mother antisocial behavior (5 mo) ^2^	0.10 (0.07)	0.19 (0.11)	0.02 (0.10)	−0.10 (0.17)	−0.29 (0.08) ***	0.14 (0.10)	−0.30 (0.11) **	0.08 (0.05)	0.11 (0.03) ***
	Father antisocial behavior (5 mo) ^2^	0.13 (0.06) *	0.25 (0.10) **	0.20 (0.09) *	0.19 (0.16)	−0.27 (0.07) ***	0.19 (0.09) *	−0.15 (0.10)	0.04 (0.05)	0.01 (0.03)
	Family configuration (5 mo) ^2^	0.09 (0.08)	0.24 (0.13)	0.07 (0.11)	0.22 (0.19)	−0.12 (0.09)	0.15 (0.11)	0.01 (0.13)	−0.14 (0.06) *	0.03 (0.04)
	Family income (5 mo) ^2^	0.19 (0.08)*	0.17 (0.13)	0.16 (0.12)	−0.19 (0.20)	0.01 (0.09)	0.03 (0.11)	−0.1 (0.13)	0.06 (0.06)	0.06 (0.04)
	Maternal education (5 mo) ^2^	0.27 (0.09) **	0.47 (0.15) ***	0.34 (0.13) **	0.49 (0.22) *	−0.44 (0.10 ***)	0.32 (0.13) **	0.24 (0.15)	0.02 (0.07)	0.13 (0.04) **
	Paternal education (5 mo) ^2^	0.05 (0.08)	−0.16 (0.12)	0.08 (0.11)	−0.03 (0.19)	0.10 (0.09)	−0.18 (0.11)	−0.30 (0.12) **	0.00 (0.06)	0.17 (0.04) ***
	Gestational smoking or substance use (5 mo) ^2^	0.04 (0.06)	0.32 (0.11) **	0.04 (0.09)	0.08 (0.16)	−0.30 (0.07) ***	0.28 (0.09) **	−0.36 (0.10) ***	0.16 (0.05) ***	0.09 (0.03) **
	Child neurocognitive skills (5 mo) ^4^	0.06 (0.06)	0.04 (0.09)	−0.13 (0.08)	−0.14 (0.14)	0.00 (0.07)	0.00 (0.08)	0.17 (0.09)	0.00 (0.04)	−0.06 (0.03) *
	Child temperament problems (1.5 years) ^2^	0.08 (0.06)	−0.04 (0.09)	−0.01 (0.08)	−0.50 (0.14) ***	−0.02 (0.07)	−0.02 (0.08)	−0.02 (0.09)	−0.03 (0.04)	0.09 (0.03) ***
	Child screen time (12 years) ^2^	0.07 (0.06)	0.21 (0.09) *	0.13 (0.08)	0.36 (0.14) **	−0.02 (0.07)	0.03 (0.08)	−0.14 (0.09)	0.01 (0.04)	−0.04 (0.03)
	Family dysfunction (12 years) ^2^	0.04 (0.06)	0.25 (0.10) **	0.11 (0.09)	0.53 (0.15) ***	−0.21 (0.07) **	0.23 (0.08) **	−0.29 (0.10) **	0.28 (0.05) ***	−0.01 (0.03)
	Adjusted R^2^	0.050 ***	0.061 ***	0.029 ***	0.030 ***	0.106 ***	0.037 ***	0.067 ***	0.051 ***	0.130 ***

Notes. * *p* ≤ 0.05, ** *p* ≤ 0.01, and *** *p* ≤ 0.001. ^1^ Teacher-reported. ^2^ Parent-reported. ^3^ Child self-reported. ^4^ Trained examiner. Analyses corrected for attrition bias. Data were compiled from the final master file of the Quebec Longitudinal Study of Child Development (1998–2013), © Gouvernement du Québec, Institut de la statistique du Québec.

**Table 5:** Unstandardized regression coefficients (standard error) reflecting the adjusted relationship between maternal depressive symptoms at age 5 months and bio-psycho-social outcomes at age 15 years.

		Age 15 Years				
	Variables	Sleep Length ^2^	Victimization ^3^	Student–Teacher Relationship ^3^	Consequential Interactions with the Child ^2^	Coercive Interactions with the Child ^2^
**Boys**	Maternal depressive symptoms (5 mo) ^2^	−0.08 (0.02) ***	0.11 (0.03) ***	0.05 (0.04)	−0.06 (0.03) *	0.04 (0.02) **
	Mother antisocial behavior (5 mo) ^2^	0.06 (0.05)	0.11 (0.109)	−0.02 (0.14)	−0.28 (0.10) **	0.03 (0.05)
	Father antisocial behavior (5 mo) ^2^	−0.07 (0.04)	0.08 (0.08)	0.15 (0.12) **	0.04 (0.09)	0.05 (0.05)
	Family configuration (5 mo) ^2^	−0.05 (0.06)	0.20 (0.11)	0.46 (0.16)	−0.16 (0.12)	−0.04 (0.06)
	Family income (5 mo) ^2^	0.02 (0.05)	−0.03 (0.10)	0.22 (0.15)	−0.07 (0.11)	0.08 (0.06)
	Maternal education (5 mo) ^2^	0.18 (0.06) **	−0.20 (0.12)	0.22 (0.17)	−0.07 (0.13)	0.04 (0.07)
	Paternal education (5 mo) ^2^	0.04 (0.05)	0.12 (0.10)	0.01 (0.15)	−0.19 (0.11)	−0.04 (0.06)
	Gestational smoking or substance use (5 mo) ^2^	0.04 (0.05)	−0.16 (0.09)	0.09 (0.13)	−0.46 (0.10) ***	0.11 (0.05) **
	Child neurocognitive skills (5 mo) ^4^	0.03 (0.04)	0.02 (0.08)	0.12 (0.11)	0.10 (0.09)	0.03 (0.04)
	Child temperament problems (1.5 years) ^2^	0.09 (0.04) *	−0.15 (0.08)	−0.16 (0.12)	−0.10 (0.09)	−0.01 (0.04)
	Child screen time (15 years) ^2^	−0.05 (0.06)	0.38 (0.12) **	−0.03 (0.17)	−0.16 (0.13)	−0.07 (0.07)
	Family dysfunction (15 years) ^2^	−0.09 (0.04) *	0.13 (0.08)	0.14 (0.12)	−0.17 (0.09) *	0.20 (0.04) ***
	Adjusted R^2^	0.034 ***	0.029 ***	0.022 ***	0.057 ***	0.033 ***
**Girls**	Maternal depressive symptoms (5 mo) ^2^	−0.06 (0.02) ***	0.03 (0.03)	−0.12 (0.05) **	−0.11 (0.04) **	0.01 (0.02)
	Mother antisocial behavior (5 mo) ^2^	0.05 (0.06)	0.08 (0.09)	0.10 (0.14)	−0.30 (0.11) **	0.08 (0.05)
	Father antisocial behavior (5 mo) ^2^	−0.04 (0.05)	0.13 (0.08)	0.09 (0.12)	−0.14 (0.10)	0.03 (0.05)
	Family configuration (5 mo) ^2^	0.03 (0.06)	−0.02 (0.10)	0.24 (0.16)	0.00 (0.13)	−0.13 (0.06) *
	Family income (5 mo) ^2^	0.01 (0.06)	0.32 (0.10) **	0.30 (0.16) *	−0.24 (0.13)	0.10 (0.06)
	Maternal education (5 mo) ^2^	0.11 (0.07)	0.01 (0.12)	0.40 (0.18) *	0.23 (0.15)	0.02 (0.07)
	Paternal education (5 mo) ^2^	−0.06 (0.06)	0.12 (0.10)	−0.08 (0.15)	−0.30 (0.12) *	−0.01 (0.06)
	Gestational smoking or substance use (5 mo) ^2^	0.02 (0.05)	0.12 (0.09)	0.09 (0.13)	−0.37 (0.10) ***	0.17 (0.05) ***
	Child neurocognitive skills (5 mo) ^4^	0.07 (0.05)	0.02 (0.08)	0.08 (0.11)	0.14 (0.09)	0.02 (0.04)
	Child temperament problems (1.5 years) ^2^	0.00 (0.05)	−0.02 (0.08)	−0.04 (0.11)	−0.03 (0.09)	−0.03 (0.04)
	Child screen time (15 years) ^2^	−0.10 (0.06)	0.19 (0.10) *	0.58 (0.14) ***	−0.27 (0.12) *	0.13 (0.06) *
	Family dysfunction (15 years)^2^	−0.03 (0.05)	0.05 (0.08)	0.38 (0.12) ***	−0.08 (0.10)	0.23 (0.05) ***
	Adjusted R^2^	0.011 *	0.025 ***	0.037 ***	0.061 ***	0.043 ***

Notes. * *p* ≤ 0.05, ** *p* ≤ 0.01, and *** *p* ≤ 0.001. ^1^ Teacher-reported. ^2^ Parent-reported. ^3^ Child self-reported. ^4^ Trained examiner. Analyses corrected for attrition bias. Data were compiled from the final master file of the Quebec Longitudinal Study of Child Development (1998–2013), ©Gouvernement du Québec, Institut de la statistique du Québec.

## Data Availability

Restrictions apply to the availability of these data. Data were obtained from the Institut de la statistique du Québec and are available with the permission of the Institut de la statistique du Québec.
